# Construction and mechanical properties of boron carbide/regenerated cellulose composite fiber based on copper ammonia method

**DOI:** 10.1371/journal.pone.0339459

**Published:** 2026-01-21

**Authors:** Yin Tang, Shouwei Ban, Jing Sun, Jianhua Zheng

**Affiliations:** 1 College of Light Industry and Textiles, Qiqihar University, Qiqihar, Heilongjiang, China; 2 College of Chemistry and Chemical Engineering, Qiqihar University, Qiqihar, Heilongjiang, China; 3 Engineering Research Center for Hemp and Product in Cold Region of Ministry of Education, Qiqihar University, Qiqihar, Heilongjiang, China; 4 Department of Academic Theory Research, Qiqihar University, Qiqihar University, Qiqihar, Heilongjiang, China; National Chung Cheng University, Taiwan & Australian Center for Sustainable Development Research and Innovation (ACSDRI), AUSTRALIA

## Abstract

The fabrication and mechanical properties of boron carbide/regenerated cellulose composite fibers prepared via the copper ammonia method were investigated. The process involves dissolving cellulose in a copper ammonia solution, incorporating boron carbide powder, and subsequently performing wet spinning to produce composite fibers. The results indicate that the addition of boron carbide significantly alters the surface morphology and color of the fibers, rendering them rougher and darker. EDS analysis confirms the presence and distribution of boron carbide within the composite fibers, while TG analysis reveals enhanced thermal stability with increasing boron carbide content. XRD and FTIR analyses provide detailed insights into the crystal structure and functional groups of the composite fibers, confirming the successful incorporation of boron carbide into the cellulose matrix. Mechanical property evaluations show that smaller boron carbide particles (500 nm) have a more pronounced detrimental effect on the fracture strength of the composite fibers, likely due to agglomeration and the formation of stress concentrations. The post-treatment with glutaraldehyde at optimal concentrations and reaction times can enhance the tenacity of the composite fibers.

## Introduction

Regenerated cellulose (RC) is a material derived from the dissolution and subsequent regeneration of natural cellulose, often utilizing solvents like copper ammonia solution [1,2], CS_2_ [3], 4-Methylmorpholine N-oxide (NMMO) [4,5] or ionic liquids [6] to achieve desired properties. It encompasses various forms such as fibers [7,8], films [9], hydrogels [10], and aerogels [11], which find applications in packaging, textiles, biomedical engineering, and wastewater treatment [12]. Recent research progress has demonstrated the application of green solvents and the development of composites with improved properties, thereby reinforcing their viability as sustainable substitutes for traditional materials [13,14].

The copper-ammonia process for fiber production involves dissolving cellulose in a copper ammonia solution (typically a mixture of copper hydroxide and aqueous ammonia) to form a viscous spinning dope, which is then extruded through spinnerets into a coagulation bath to regenerate cellulose fibers [12]. The process is particularly suitable for fibers requiring gentle processing at lower temperatures, as it avoids polymer degradation. Copper ammonia cellulose fibers also exhibit functional versatility. For instance, they can be integrated into composite materials for applications like dye adsorption and antimicrobial membranes by combining with polymers such as polyacrylonitrile (PAN) [15]. Additionally, copper ammonia fabrics serve as flexible substrates for advanced materials, such as multilayer conductive composites (e.g., CF/PPy/Cu), which demonstrate high electromagnetic shielding effectiveness (30–50 dB) due to synergistic wave-absorption and reflection properties [16].

Boron carbide (B_4_C) is a ceramic material that is highly regarded for its exceptional properties, including a high melting temperature, outstanding hardness, low density, favorable mechanical strength, significant neutron absorption capacity, and strong chemical stability. These characteristics make it well-suited for use in high-temperature thermoelectric systems, lightweight protective armor, fast neutron reactors, nuclear reactor neutron absorption, as well as abrasive applications [17,18]. Boron carbide composites are materials that combine boron carbide with other substances to enhance or impart new properties [19–21]. Boron carbide composite fibers are synthesized through methods such as chemical vapor deposition (CVD) [22], melt-spinning processes [23] or composite [24,25] etc. Compared with the traditional manufacturing process of B_4_C structural materials, which usually requires high temperatures (about 2000°C), the synthesis of B_4_C composite fibers requires lower temperatures and can form fine and continuous fibers. Meanwhile, the special characteristics of B_4_C, such as its high hardness and unique properties as a nonoxide ceramic, are preserved and utilized in this fiber formation process. Kakiage & Kobayashi [26] presents a method for fabricating boron carbide fibers through carbothermal reduction using electrospinning. The process involved preparing condensed boric acid (H_3_BO_3_)-poly(vinyl alcohol) (PVA) product fibers via electrospinning from a H_3_BO_3_-PVA/dimethyl sulfoxide solution with added glycerin. These fibers were then thermally decomposed in air to form a fibrous B_4_C precursor consisting of boron oxide and carbon components. The precursor was heat-treated at 1400 °C in an Ar flow to produce the crystalline B_4_C fibers. The advantages of this method include the use of conventional carbothermal reduction techniques, the ability to synthesize B_4_C fibers at relatively low temperatures (1200∼1400 °C). Rallini et al. [24] investigated the impact of boron carbide nanoparticles on the fire resistance of carbon fiber/epoxy composites. They prepared the composites by mechanically stirring and ultrasonically treating the nanoparticles in the epoxy matrix, achieving good dispersion without increasing viscosity or compromising mechanical properties. The boron carbide nanoparticles, due to their unique chemical reactions with oxygen and combustion by-products at high temperatures, hold particular promise in creating a protective layer that inhibits carbon fiber oxidation and acts as a high-temperature adhesive. The resulting composites exhibited enhanced thermal stability and fire resistance, with a 5% boron carbide concentration showing the most significant benefits.

Most reported B_4_C/polymer composites utilize synthetic matrices and often require processing conditions or result in rigid composites unsuitable for flexible textile applications [27–29]. Cellulose composites are widely used in the electronic field. They can be fabricated into flexible energy harvesting devices through 3D printing, and also serve as efficient thermal interface materials for heat dissipation in electronic devices [30–32]. Moreover, they possess electromagnetic shielding properties, making them suitable for microelectronic packaging requirements. At the same time, their environmental friendliness also holds potential in areas such as bio-packaging. Boron combined with cellulose can not only significantly enhance the thermal conductivity of composite materials, build an efficient thermal conduction network to meet the heat dissipation requirements of electronic devices, but also improve the mechanical strength, flexibility and thermal stability of the materials, while maintaining good electrical insulation, providing support for the development of multifunctional composite materials [33,34].

In this paper, a boron carbide/regenerated cellulose (B4C/RC) composite fiber was constructed by the copper-ammonia method, and its structure and mechanical properties were characterized. Its preparation process was carried out at room temperature. In contrast, regenerated cellulose offers a renewable, biodegradable, and versatile matrix that can be processed into various forms, including fibers. The copper-ammonia method provides a unique low-temperature, aqueous pathway for fabricating such composite fibers, starkly contrasting with the high-temperature sintering typically required for B_4_C ceramics or the melt-spinning of synthetic polymers. This material is expected to combine the flexibility, water absorption, and moisture permeability of cellulose fibers, as well as the neutron absorption characteristics of boron carbide. Regenerated cellulose possesses flexibility, degradability and potential for multi-form processing, while B_4_C boasts high-efficiency neutron absorption and high-temperature resistance. The copper-ammonia method offers a low-temperature and green preparation route for the combination of the two. This makes B4C/RC composite fibers have exploration value in fields such as nuclear radiation shielding, aerospace, and biomedicine, where there are special requirements for material performance. The B4C/RC composite fibers are expected to possess excellent boron-carrying capacity, thereby providing high neutron shielding efficiency. These fibers can be utilized as textile raw materials and show significant application potential across various industries, such as nuclear power, aerospace, and healthcare.

## Experimental

### Reagents

Cupric ammonia solution, Cu^2+^ = 1±0.02 mol/L, Fupait Environment Technology (Suzhou) Co., Ltd.; Absorbent cotton, Shandong Brilliance Medical Equipment Co., Ltd.; Citric acid, purity ≥ 99.5%, Shanghai Macklin Biochemical Co., Ltd.; Boron carbide powder, purity ≥ 97%, 500 nm 1000 nm, purchased from Nangong Xinhu Wear-resistant Electrode Factory; Sodium hydroxide, 95%, Shanghai Macklin Biochemical Co., Ltd.; Sulfuric acid, AR, Sinopharm Chemical Reagent Co., Ltd.

### Construction of boron carbide/regenerated cellulose composite fiber

**Fig 1:**
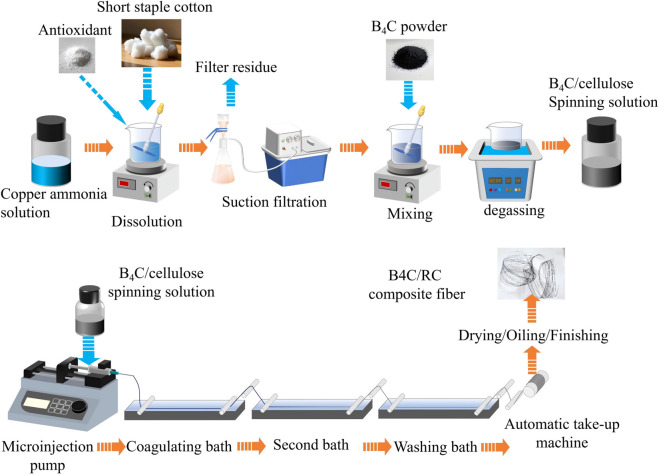
Construction process of B4C/RC.

After 500 mL copper ammonia solution and 100 g absorbent cotton were mixed and stirred, the solid was fully dissolved, 5% citric acid was added, and the copper ammonia pulp was filtered by polypropylene filter with an average pore size of 1∼20μm. The pulp was divided into the same parts with equal weight and mixed with 500 nm boron carbide powder (with the addition amount ranging from 0 to 0.5 wt%) to form boron carbide/copper ammonia pulp mixture. Using ultrasonic degassing for 20 min, the inner bubble was removed as far as possible, and the boron carbide/cellulose spinning solution was obtained. The spinning liquid was added to the micro-injection pump, the injection pump was opened, the spinning liquid was extruded into the coagulation bath through the spinneret, and the spinning liquid was quickly drawn and solidified. The fibers were collected after 20% sodium hydroxide coagulation bath, 3% sulfuric acid second bath and washing bath. The B4C/RC composite fiber was prepared after oiling and drying (Fig 1).

### Structural characterizations of B4C/RC composite

The microstructure and morphology of composites were characterized using field-emission scanning electron microscopy (FE-SEM, Zeiss G300, Carl Zeiss AG, USA). Compositional analysis was performed by energy-dispersive X-ray spectroscopy (EDS, xplore30, Oxford Instruments, UK). The Thermal Gravimetric Analyzer (TGA) measurement of the materials (no paraffin contained) was conducted by the TG analyzer (STA 449A, Netzsch, Germany) in the nitrogen atmosphere. X-ray photoelectron spectroscopy (XPS) spectra were acquired using a Thermo Fisher Scientific EscaLab 250Xi X-ray Photoelectron Spectrometer.

## Results and discussion

### Microstructure analysis of materials

**Fig 2:**
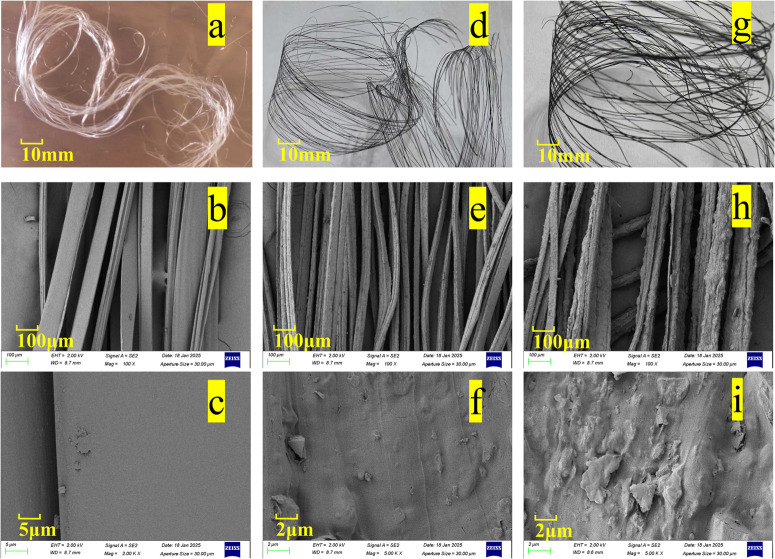
Macroscopic and microscopic morphology of B4C/RC fibers. (a)(b)(c) 0 boron carbide addition; (d)(e)(f) the mass fraction of boron carbide is 10%; (g)(h)(i) the mass fraction of boron carbide is 30%.

Fig 2 shows the macro and micro morphologies of B4C/RC composite fibers with different B_4_C additions. Macroscopically, the color transition from colorless/white to dark gray (Fig 2a, 2d and 2g) visually confirms the successful combination of boron carbide (B_4_C) with cellulose. SEM images (Fig 2b, 2c, 2e, 2f, 2h and 2i) reveal that the pristine RC fiber exhibits a smooth surface, whereas the incorporation of B_4_C introduces significant surface roughness and particulate features, which become more pronounced with higher B_4_C content. This increased surface roughness, caused by the protrusion of hard B_4_C particles, suggests a potential compromise in mechanical properties. These surface irregularities can act as stress concentration sites, initiating micro-cracks under tensile load and leading to reduced fracture strength [35]. The absence of large-scale particle debonding in the micrographs indicates some level of physical adhesion between B_4_C and the cellulose matrix, although the inherent weakness of this filler-matrix interface likely remains a key factor governing the mechanical performance.

### Boron distribution and thermal stability analysis of B4C/RC fibers

**Fig 3:**
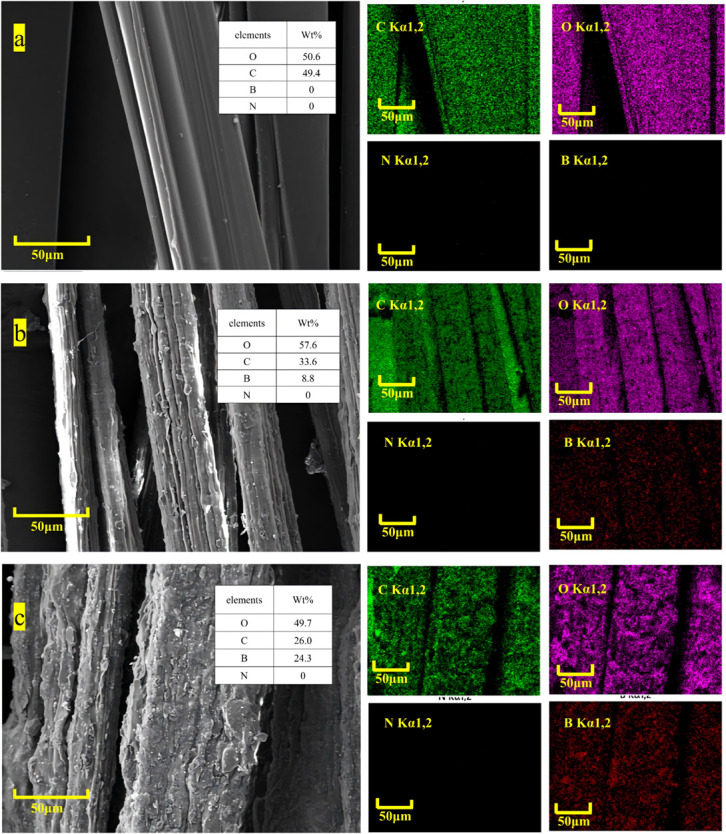
Macroscopic and microscopic morphology of B4C/RC fibers. (a)(b)(c) 0 boron carbide addition; (d)(e)(f) the mass fraction of boron carbide is 10%; (g)(h)(i) the mass fraction of boron carbide is 30%.

In order to specifically understand the retention and distribution of boron carbide in B4C/RC fiber, EDS analysis of the material was performed, as shown in Fig 3. The distribution of C, O, N and B is obviously different in the fibers with different boron carbide content. Since the main base of the composite fiber is regenerated cellulose, the distribution of C and O elements is almost the same as the SEM morphology of the fiber. Fig 3a shows the regenerated cellulose fiber with 0 boron carbide added, and almost no element B is analyzed. Fig 3b shows the B4C/RC composite fiber with 10% boron carbide added, and a small amount of B element can be seen on the surface of the fiber. Compared with the composite fiber with the addition amount of 30%, as shown in Fig 3c, the distribution of element B on the surface of the material is significantly increased, and the distribution of element B is uniform. The EDS pattern of element B is similar to that of element C or O, and the outline of the composite fiber can be clearly seen.

**Fig 4:**
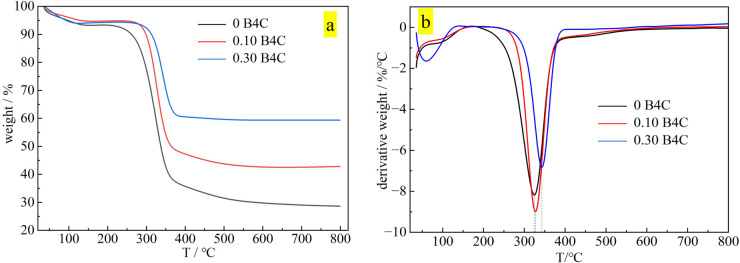
TGA (a) and DTG (b) analysis of B4C/RC fibers.

Furthermore, TGA analysis of the composite fiber was performed to understand the operating temperature range of the composite fiber, as shown in Fig 4. The thermal stability of the material during hot working is evaluated and further analysis of the boron carbide load is performed. With the increase of boron carbide addition, the residual quality of composite fiber also increases. Compared with the regenerated cellulose with a boron carbide content of 0 and the B4C/RC fibers with boron carbide contents of 10% and 30%, the residual mass is positively correlated with the boron carbide content. The difference in residual mass can be used to characterize its content. The DTG analysis shows that when the addition amount of B4C changes from 0 to 30%, the maximum degradation rate of the material increases from 320 °C to 348 °C. With the increase of the B4C proportion, the thermal stability of the material is enhanced accordingly.

## Results and discussion

### XRD Analysis of B4C/RC fibers

**Fig 5:**
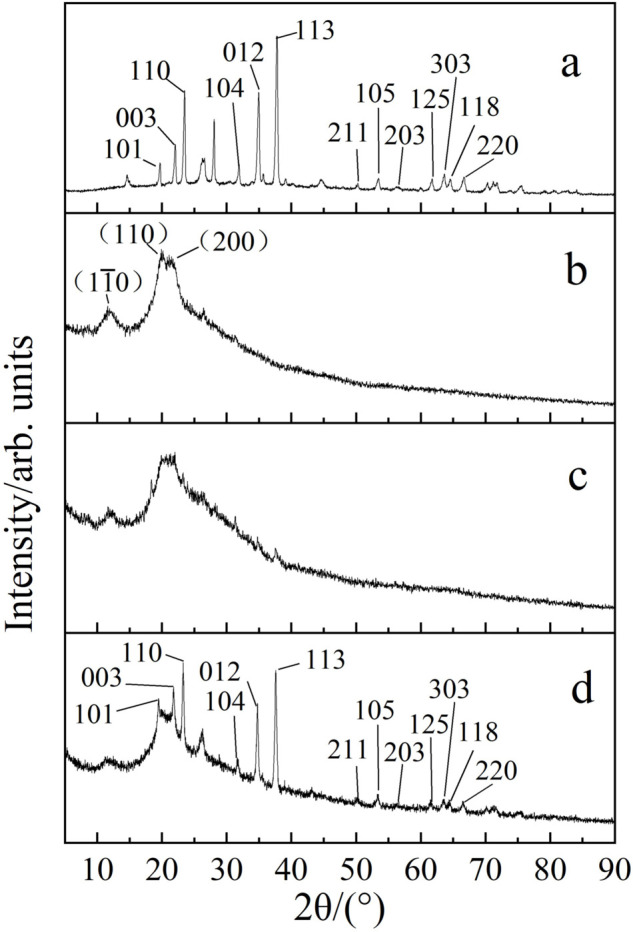
XRD analysis of B4C/RC fibers. (a) boron carbide powder; (b) 0 boron carbide addition; (c) the mass fraction of boron carbide is 10%; (d) the mass fraction of boron carbide is 30%.

Fig 5a corresponds to the XRD crystal pattern of the raw boron carbide particles. Theta = 19.7 ° 2 (101), 22.1 ° (003), 23.5 ° (110), 31.9 ° (104), 34.9 ° (012), 37.8 ° (113), 50.3 ° (211) and 53.48 ° (105), 56.3 ° (203), 61.8 ° (125), 63.7 ° (303), 64.6 ° (118), 66.7 ° (220). A small amount of carbon and boric acid residues can be observed. It can be seen from Fig 5b that the diffraction peaks of the regenerated cellulose fibers obtained in this experiment 2*θ* = 12.0, 20.0 ° and 22.5° correspond to the crystal faces of the cellulose cell (11$\overset{―}{0}$), (110) and (200), which can be seen to belong to the crystal type of cellulose II. The B4C/RC fiber prepared in this experiment increased with the addition of boron carbide, as shown in Fig 5c and 5d. The characteristic peaks of these boron carbide particles became more obvious, and the strength increased with the increase of the addition amount. Boron carbide is compounded in the form of physical doping into the regenerated cellulose.

This change is positively correlated with the change in the content of B4C, but not linearly. Although the samples were processed such as shearing and grinding before the XRD test, some B4C may still be wrapped by regenerated cellulose and fail to show the characteristic diffraction peaks in the XRD pattern. When the addition amount of B4C is too high, B4C particles are exposed on the surface of the material, and the related characteristic diffraction peaks of B4C are accordingly displayed in the spectrum.

### Infrared spectrum analysis of B4C/RC fibers

**Fig 6:**
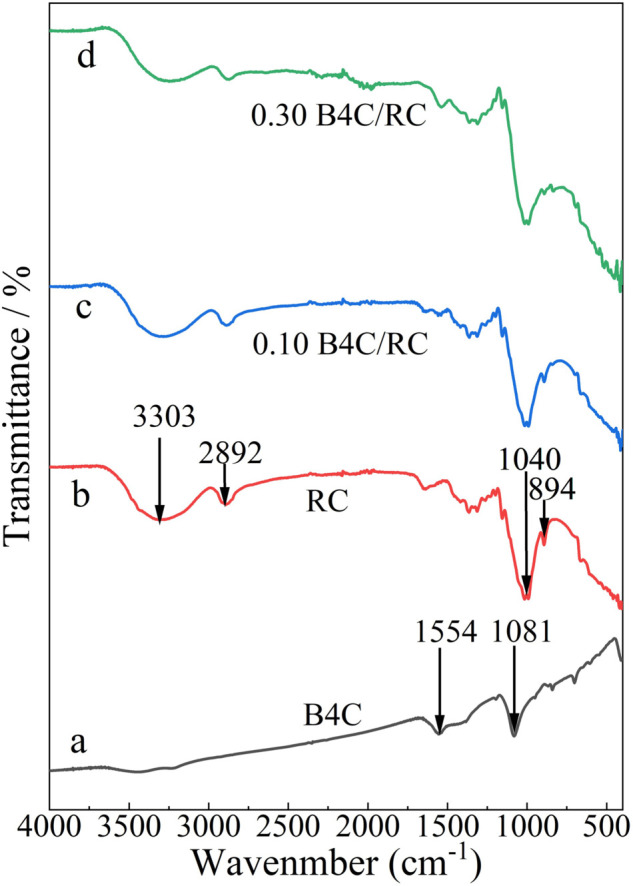
FTIR analysis of B4C/RC fibers. (a) boron carbide powder; (b) 0 boron carbide addition; (c) the mass fraction of boron carbide is 10%; (d) the mass fraction of boron carbide is 30%.

The FTIR spectrum of the raw B_4_C shows several bands: a band appeared at 1081 cm^−1^ may be attributed to B-B linkage, as shown in Fig 6. Band positioned at 1554 cm^−1^ may be attributed to free carbon atoms in the B_4_C or C-B-B linear chains that interlock. As shown in curve b of Fig 6, 3303 cm^−1^, 2892 cm^−1^, and 1040 cm^−1^ are the characteristic peaks of the stretching vibrations of O-H, C-H, and C-O bonds of regenerated cellulose, respectively. The 894 cm^−1^ band is very sensitive to the amount of crystalline and amorphous structure of cellulose. Here the band becomes a sharp narrow peak, which also proves from the side that the synthesized fiber is mainly cellulose II. The addition of boron carbide makes the composite different in infrared characteristics. The overlap between the strong absorption band at 1040 cm^−1^ and the absorption peak at 1081 cm^−1^ of boron carbide is difficult to distinguish, and the strength at 1554 cm^−1^ of boron carbide is obviously strengthened with the increase of its addition amount. The FTIR spectral evolution suggests potential interfacial interactions between boron carbide and the cellulose matrix, beyond mere physical blending. With increasing B_4_C content, the broad O–H stretching band centered at 3303 cm^−1^ exhibits gradual broadening and a slight red shift. It is possible that the coordination of electron-deficient boron atoms (on the B_4_C surface) with electron-rich oxygen atoms present in cellulose’s hydroxyl groups. The formation of these weak coordination bonds reduces the O–H bond force constant, manifesting as red shift and broadening. Concurrently, the spectral complexity in the 1040–1100 cm^−1^ region arises not only from the overlap of the cellulose C–O stretching (1040 cm^−1^) and the B_4_C B–B vibration (1081 cm^−1^) but also from potential perturbations to the C–O vibrational modes induced by these interfacial interactions. These findings indicate the presence of favorable interfacial chemistry at the B_4_C-cellulose junction, which could contribute to the enhanced thermal stability observed in TG analysis by restricting segmental mobility of cellulose chains.

### Mechanical property analysis and strengthening of B4C/RC fibers

**Fig 7:**
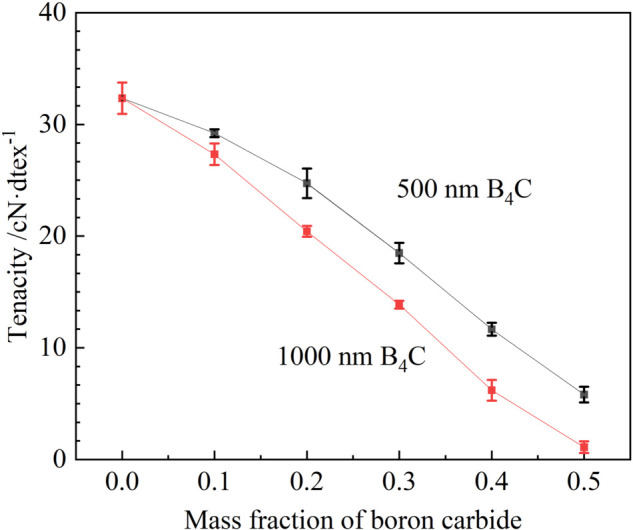
The influence of the particle size and addition amount of boron carbide on the mechanical properties of B4C/RC fibers.

As the doping amount of particles increases, the mechanical tolerance performance of the material usually decreases accordingly [36,37]. To investigate the influence of the composite incorporation of boron carbide on the mechanical properties of the material, the fracture strength of the samples was tested. When the particle size of boron carbide is excessively large (≥1000 nm), poor dispersion in the copper ammonia spinning solution leads to agglomeration, which can readily block spinneret orifices, disrupt the continuity of wet spinning, and increase surface defects in the resulting fibers. Conversely, using boron carbide particles that are too fine (≤300 nm) presents different challenges: such ultrafine powders typically require specialized synthesis methods, resulting in significantly higher costs compared to 500 nm particles. All tests were conducted at a rate of 0.5 mm/min. Five parallel experiments were carried out for each test. The gauge length was 25×10^−3^ m. Fig 7 shows the influence of the addition amount of boron carbide and the addition of boron carbide with different particle sizes (500 nm and 1000 nm) on the fracture strength of regenerated cellulose fibers. Pure regenerated cellulose fibers usually have relatively good tensile strength [38,39]; the regenerated cellulose prepared in this paper has a tensile strength of up to 32.34 cN/dtex. Along with the increase in the mass fraction of B_4_C, both particle sizes of B_4_C resulted in a significant decrease in fracture strength. Under the same addition amount of boron carbide, the fracture strength of the composite fibers prepared with 500 nm particle size boron carbide is always higher than that with 1000 nm particle size. Smaller particle sizes exhibit better mechanical properties due to better dispersion and interfacial bonding strength. B_4_C is an inorganic ceramic particle characterized by a chemically inert surface, whereas regenerated cellulose is an organic polymer featuring abundant surface-exposed hydroxyl groups (-OH). The substantial disparity in their chemical nature leads to limited interfacial adhesion between the two materials. It is challenging to establish strong chemical bonds or effective adsorption through physical blending alone, resulting in inadequate interfacial bonding between B_4_C particles and the cellulose matrix. During tensile loading, interfacial debonding between the particles and the matrix is likely to occur, leading to void formation and further degradation of the mechanical properties of the fibers. Although the red shift of the O-H bond observed in FTIR analysis indicates the presence of weak coordination, the strength of this interaction is insufficient to compensate for the detrimental effects arising from poor interfacial bonding. Therefore, a particle size of 500 nm represents an optimal compromise between process feasibility and cost-effectiveness. Furthermore, an attempt was made to compare the effect of adding and not adding the surfactant sodium dodecyl sulfate (SDS) on the dispersion performance of boron carbide. Mixing and degassing after adding SDS can slightly improve the dispersion of boron carbide, but it has no significant impact on the mechanical properties of the material. This requires further analysis.

**Fig 8:**
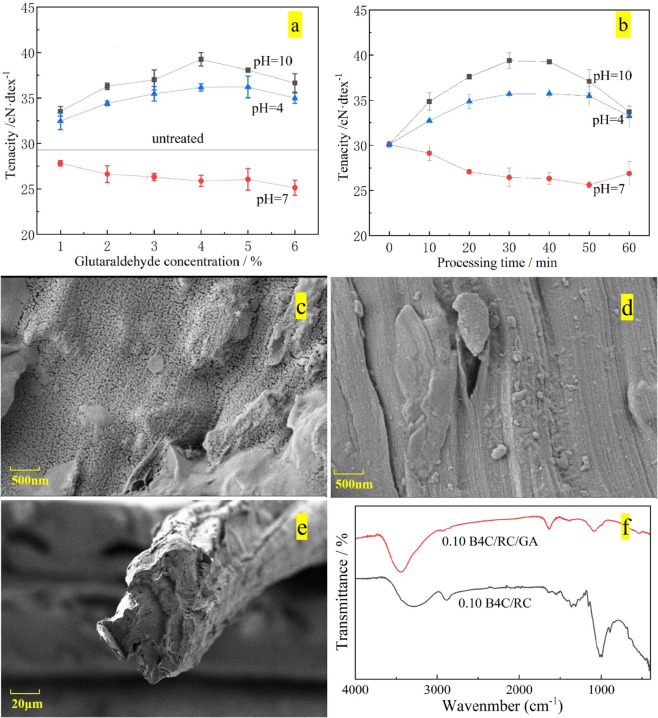
The influence of glutaraldehyde treatment on the mechanical properties of B4C/RC composites fibers. (a) The influence of glutaraldehyde concentration; (b) The influence of processing time; (c) SEM microstructure of unmodified B4C/RC (0.10 B4C/RC); (d) SEM microstructure of B4C/RC modified with glutaraldehyde (0.10 B4C/RC/GA); (e) The cross-sectional morphology of B4C/RC fiber modified with glutaraldehyde; (f) Comparison of infrared spectra of composite fibers before and after glutaraldehyde modification treatment.

In order to further improve the mechanical properties, the mechanical properties of the samples treated with glutaraldehyde were tested. Fig 8a and Fig 8b respectively show the variation of the fracture strength of B4C/RC composites with the concentration of glutaraldehyde and the treatment time under different pH values. The modification of B4C/RC composites with glutaraldehyde in acidic and alkaline environments is conducive to improving the mechanical properties of the materials. At pH = 4 and pH = 10, tenacity showed a trend of first increasing and then decreasing. A moderate concentration of glutaraldehyde (about 4% at pH = 4 and about 3% at pH = 10) and treatment time (about 30 minutes) could improve the mechanical properties. However, when pH = 7, the tenacity of B4C/RC composites showed a downward trend. It can be seen from the SEM images that the surface of the unmodified B4C/RC fibers is relatively loose (Fig 8c), with some boron carbide exposed on the surface, presenting a porous structure. In contrast, the surface of the modified B4C/RC is compact (Fig 8d), with almost all the boron carbide encapsulated within the fiber network. The cross-section of the fibers shows a rough and multi-creased layered structure without obvious brittle fracture surfaces (Fig 8e). This morphology indicates that when the material fractures under force, the energy is effectively dispersed through the deformation of the multi-layer structure and the interface interaction, demonstrating the synergistic strengthening effect between the organic and inorganic phases after modification.

Further analysis of the material structure was conducted through infrared spectroscopy (Fig 8f). The range of 1310 to 1361 cm^−1^ corresponds to the bending vibration peaks of hydroxyl groups in cellulose, and its intensity decreases as the number of hydroxyl groups reduces. A strong absorption peak appears in the 1634 cm^−1^ range, which is a typical signal of the reaction between aldehyde groups and hydroxyl groups in cellulose. Cellulose originally has a broad and strong hydroxyl (-OH) stretching vibration peak at 3200 3400 cm^−1^. After modification, due to the reaction and consumption of hydroxyl groups with aldehyde groups, the intensity of this peak will significantly decrease and the peak shape will also narrow. These changes indicate the successful modification of B4C/RC by glutaraldehyde. For a more convenient understanding, the properties of the raw materials regenerated cellulose and B4C/RC are compared, as shown in Table 1.

**Table 1:** Performance comparison of raw materials regenerated cellulose and B4C/RC composites.

Performance categories	Mechanical properties	Thermal properties	Other key properties
Pure regenerated cellulose fiber	It has excellent fracture strength, reaching up to 32.34 cN/dtex	It has relatively low thermal stability, with a maximum degradation temperature of 320°C and little residual mass	Smooth surface; No element B; The XRD pattern shows the standard cellulose II crystal form.
B4C/RC composite material fibers	The fracture strength decreases significantly with the addition of B_4_C. However, after treatment with glutaraldehyde under the optimal conditions, its toughness can be effectively improved (such as in acidic/alkaline environments, with specific concentrations and treatment times).	The thermal stability has been significantly enhanced; For instance, when the addition amount of B_4_C is 30%, the maximum degradation temperature rises to 348°C, and the residual mass increases with the addition amount.	The surface is rough due to the embedding of B_4_C particles. EDS confirmed the uniform distribution of element B. XRD and FTIR indicated that B_4_C was successfully physically doped and there were potential interactions at the interface.

## Conclusion

The B4C/RC composite fibers were successfully synthesized via the copper ammonia method in this study. The incorporation of boron carbide into the cellulose matrix was confirmed through various analytical techniques, including FE-SEM, EDS, TG, XRD, and FTIR. The results demonstrate that the addition of boron carbide significantly influences the microstructure, thermal stability, and mechanical properties of the composite fibers. While the mechanical properties are compromised with higher boron carbide content, particularly with smaller particle sizes, the application of a suitable glutaraldehyde post-treatment can effectively mitigate these effects and improve the tenacity of the composite fibers.

Regenerated cellulose possesses flexibility, degradability and potential for multi-form processing, while B_4_C boasts high-efficiency neutron absorption and high-temperature resistance. The copper ammonia method offers a low-temperature and green preparation route for the combination of the two. This makes B4C/RC composite fibers have exploration value in fields such as nuclear radiation shielding, aerospace, and biomedicine, where there are special requirements for material performance. The composite fibers demonstrated excellent boron loading capacity and mechanical performance, indicating their potential as textile raw materials for applications in nuclear power, aviation, and healthcare. Future research could focus on optimizing the boron carbide dispersion within the cellulose matrix and further improving the mechanical properties of the composite fibers to expand their application scope.

## Supporting information

S1 File This is the minimal data set. (ZIP)
