# Supplementary material for: Construction and mechanical properties of boron carbide/regenerated cellulose composite fiber based on copper ammonia method
Source: PLoS One. 2026 Jan 21;21(1):e0339459. doi: 10.1371/journal.pone.0339459 (PMC12822970; doi:10.1371/journal.pone.0339459)
Supplement: S1 File — (ZIP) [file pone.0339459.s001.zip › S1 File/minimal data set/Fig.3.docx]

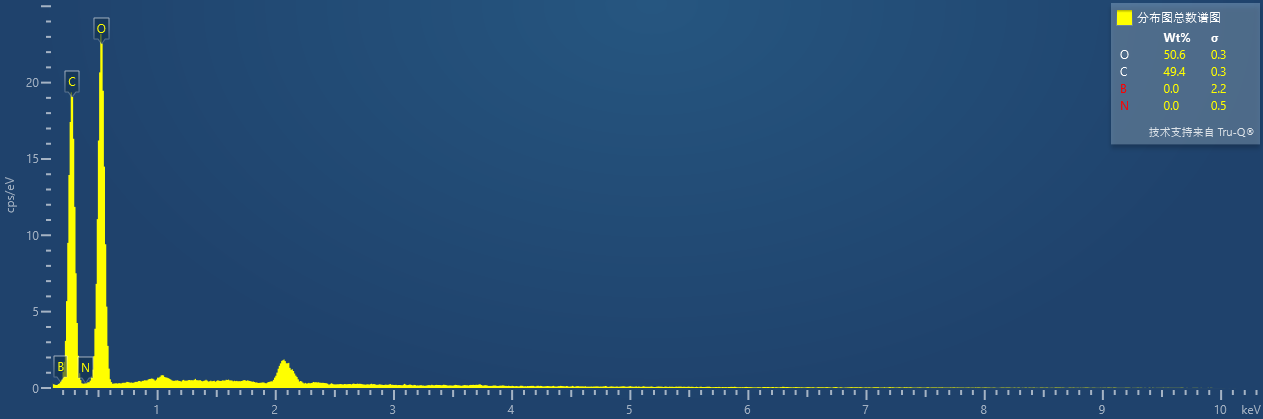


Fig.3.a EDS semi-quantitative analysis


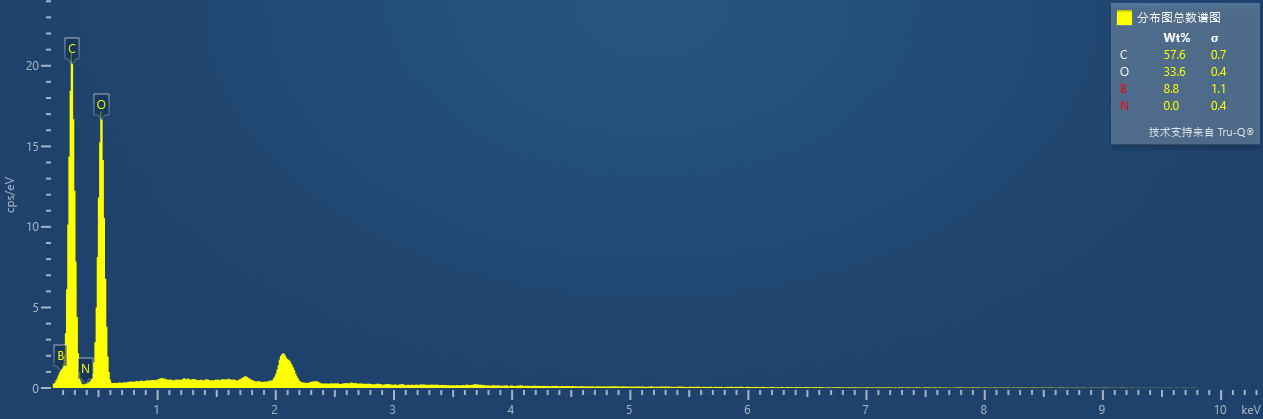


Fig.3.b EDS semi-quantitative analysis


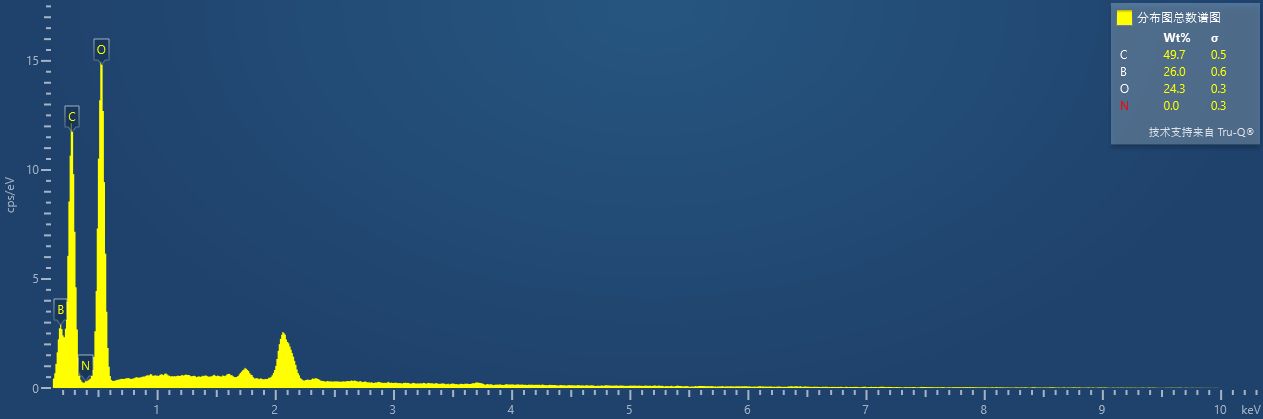


Fig.3.c EDS semi-quantitative analysis
